# Temperature measurement data gathered from off-gas duct of a submerged arc furnace utilised for silicomanganese production

**DOI:** 10.1016/j.dib.2021.107242

**Published:** 2021-06-24

**Authors:** Martin Sitefane, Joalet Dalene Steenkamp, Khutso Rampyapedi

**Affiliations:** a Mintek, Randburg 2125, South Africa; b University of the Witwatersrand, Johannesburg 0001, South Africa; c Transalloys, Emalahleni 1036, South Africa

**Keywords:** Sintering, Dust, Temperature, Off-gas duct, Blockages, Silicomanganese, Submerged arc furnace

## Abstract

Blockage of the off-gas duct by dust contained in raw off-gas extracted from a submerged arc furnace (SAF), has been a recurring problem at a South African silicomanganese (SiMn) producer. The problem experienced has prompted an investigation in which sintering of the dust is evaluated as a possible mechanism for the observed blockages. As sintering is heavily dependant on temperature, one of the key factors to the investigation was determining the typical temperatures across the areas of the duct where blockages are commonly observed. Datasets of the measurements of the duct temperatures, across two extreme points in the duct, are hereby presented. Extreme 1 (SAF burden), which represented the hottest zone, was taken on the SAF burden using a calibrated optical pyrometer. Measurements in extreme 2 (duct cap), which represented the coldest zone in the ducts, were taken using a fixed thermocouple. Measurements from both extremes were taken over a period of four days. This dataset was useful in that it defined the minimum and maximum temperatures utilised in a laboratory-scale investigation onto the potential for sintering to be the cause of duct blockages on the SAF under investigation. Furthermore, going forward this data can be used in modelling of raw gas mass and heat transfer or other related dust transportation phenomena along the ducts. Additional foreseeable uses of this data includes applications in duct and baghouse designs where dust-laden off-gas temperature is a factor, energy loss calculations for the process, and researchers or other stakeholders interested in knowing the dust-laden off-gas exit temperature for a SAF operation applying an open or semi-open roof configuration, in SiMn production.

**Specifications Table**

**Table:** 

Subject	Engineering
Specific subject area	Temperature measurement data used in evaluation of potential for sintering along the off-gas ducts of a submerged arc furnace (SAF) applied in silicomanganese production
Type of data	Tables, graphs
How data were acquired	K-type thermocouple Optical pyrometer (Minolta/Land Cyclops 52 infrared thermometer)
Data format	Raw, filtered, analysed
Parameters for data collection	For both the duct cap and SAF burden: • Maximum measured temperature in °C • Minimum measured temperature in °C • Calculated mean temperature in °C • Calculated standard deviation in °C
Description of data collection	The temperature was measured in two ways: On the SAF burden (hot section), an optical pyrometer was used to take measurements every 2 h, between 8 am – 4 pm, over four days. In the duct cap (cold section), a thermocouple was installed to take continuous measurements also over four days.
Data source location	Transalloys Emalahleni South Africa X: 29°7′32″ Y:−25°53′19″ Mintek Johannesburg South Africa GPS - 26° 5′25.77 S – 27° 58′45.27 E
Data accessibility	With the article

## Value of the Data

• The data was important for the current investigation in helping to define the temperature limits for evaluating the possibilities of sintering. It can also be used as one of the inputs during the design of SAF ducts, understanding the heat transfer along the ducts, for modelling dust flow patterns, for determination of chemical rates, and for calculating the heat losses to the dust-laden off-gas.
• With the first bullet point in mind, those who could benefit from the data include: researchers (use data for related or other research), engineers (structural design of ducts to combat the observed temperatures), plant personnel's (understand the typical temperatures encountered in such processes), ordinary learners (wishing to understand concepts related to SAF energy loss, cooling etc.).
• The data can be used to evaluate different duct designs should the main cause of blockages be temperature related. An example of this may be a duct design which include additional cooling to ensure temperatures below dust sintering temperatures.

## 1 Data Description

**Fig. 1:**
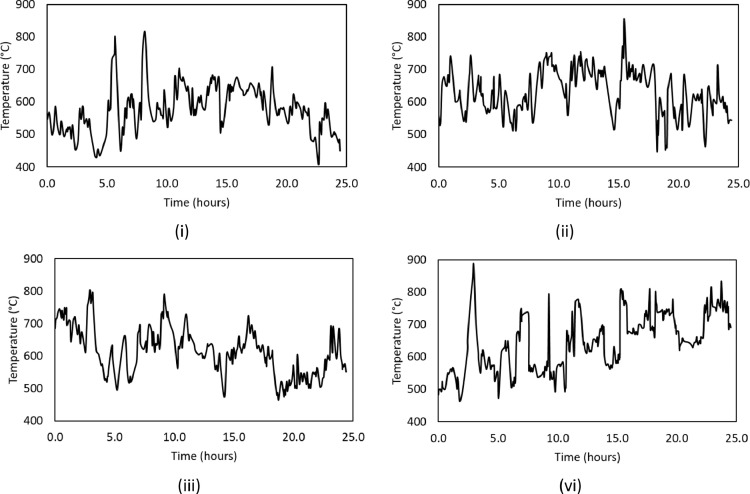
Graphical representation of change in duct cap temperatures over time, with: (i) Day 1, (ii) Day 2, (iii) Day 3, and (iv) Day 4.

The datasets presented here were obtained from direct industrial measurements, taken over four days. Each graph in Fig. 1 graphically depicts the change in duct cap temperature over a 24 hour period. The duct cap temperatures were measured using a permanently stationed K-type thermocouple. The practise of using thermocouples in high intensity environments is a common practise [1,2]. It is generally known that radiation in such environments can cause discrepancies in the measured temperature [2]. These discrepancies can be countered in several ways: the usage of a radiation shield, taking readings over a long period to obtain the average temperature with time, using smaller thermocouple beads, using new shiny thermocouples to reduce emissivity [1,^2^,3]. In this work, measurements were performed over an extended period to mitigate against radiation. Since the graphs below were reconstructed from industrial graphical images, the raw data is available in the appendix (labelled as Table II), and as a Microsoft excel sheet file named Raw data *- the actual sheet is named* (Table II).

**Fig. 2:**
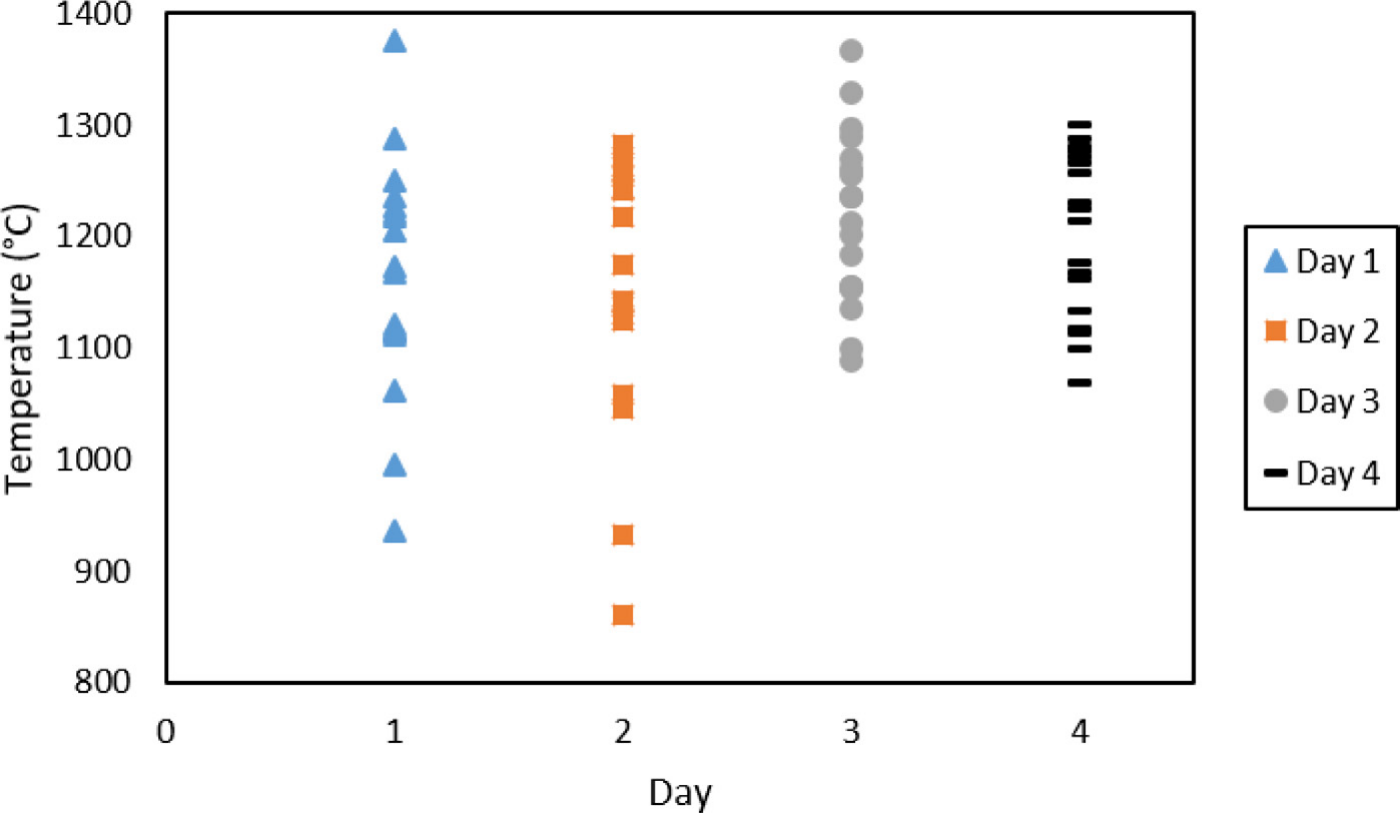
Graph reconstructed from industrial measurements of SAF burden temperatures (maximum temperature) for day 1 to day 4.

Fig. 2 graphically depicts the outcomes of the measurements of the SAF burden temperatures. The primary data from which this graph was constructed is available in the repository as a Microsoft Excel file named *Data* in the “SAF burden” sheet.

Table 1 summarises the calculated mean, calculated standard deviation, measured maximum and measured minimum temperatures of the duct cap as well as the SAF burden. It should be noted that the data for the duct cap was obtained by extracting the peak measurements across all four graphs in Fig. 1. Again the filtered data is available as a Microsoft excel file named *Data* under the “duct cap” sheet.

**Table 1:** Data summary of duct cap and SAF burden temperature measurements.

	Duct Cap (°C)	SAF burden (°C)
Mean	623	1184
Standard deviation	83	97
Maximum – measured	885	1375
Minimum - measured	408	861
Maximum – calculated	706	1281
Minimum - calculated	540	1087

## 2 Experimental Design, Materials and Methods

### 2.1 Duct cap (Cold section)

**Fig. 3:**
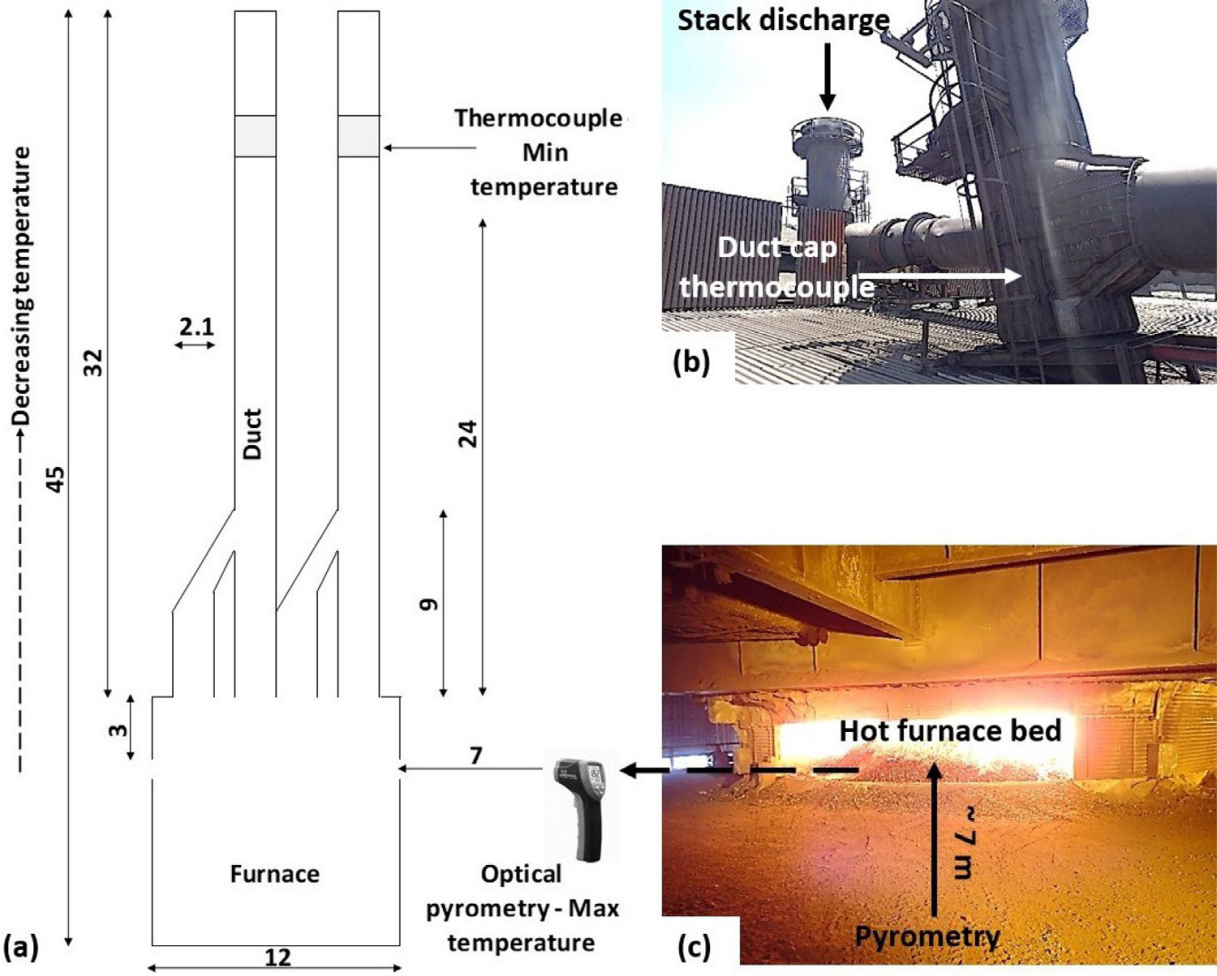
of (a) Schematic diagram showing position of duct cap and SAF burden relative to the SAF, (b) photograph showing duct cap thermocouple position, and (c) photograph showing SAF bed appearance during operations (all values in metres).

In terms of the duct cap temperature, continuous measurements were taken by a permanently installed K-type thermocouple. The thermocouple was positioned close to the tip end of the ducts, before the start of the horizontal stack section (see Fig. 3). It was placed inside the duct in such a fashion that the measurements were obtained from the dust-laden off-gas itself, instead of the steel shell temperature. A separate steel shell thermocouple was also installed. As previously mentioned, measurements were automatically logged on a continuous base. The outcome was a graphical depiction that could easily be viewed on the operator's screen inside the plant control room.

### 2.2 SAF burden (Hot section)

In the case of SAF burden temperatures, a calibrated optical pyrometer (Minolta/Land cyclops 52 infrared thermometer) was utilised. The pyrometer was set at an emissivity of 0.7 Ɛ (from personal advise and values in reference [4]), and a distance of infinity (to account for the 7 metres distance between the SAF and the point of measurement). The SAF burden was stationed about 3 metres below the lowest point of the duct. Measuring the temperature entailed pointing the pyrometer on the surrounding flames observed on the SAF bed. Measurements were taken at four randomly selected positions, between 8 am and 4 pm (every two hours), to account for variance in heat distribution across the SAF bed. Fig. 3 presents a detailed diagram as well as photographs, depicting the positions where temperature measurements were taken.

## CRediT Author Statement

**Martin Sitefane:** Conceptualization, Methodology, Formal analysis, Investigation, Data curation, Writing-Original draft preparation, Visualisation, Project administration, funding acquisition; **Joalet Steenkamp:** Writing, reviewing and editing, Supervision; **Khutso Rampyapedi:** Support, Data acquisition.

## Declaration of Competing Interest

The authors declare that they have no known competing financial interests or personal relationship which that have or could be perceived to have influenced the work reported in this article.

## Appendix Supplementary materials

Image, application 1

## References

[1] Shannon K.S., Butler B.W. (16-20 November 2003). A review of error associated with thermocouple temperature measurement in fire environments. 2nd International Wildland Fire Ecology and Fire Management Congress. Coronado Springs Resort.

[2] Shaddix C.R. (23-24 April 2017). A new method to compute the proper radiant heat transfer correction of bare-wire thermocouple measurements. 10th US Combustion Meeting. College Park.

[3] Davis J.C. (1969). Radiation Errors in Air Ducts Under Nonisothermal Conditions Using thermocouples, thermistors, and a Resistance Thermometer.

[4] The Engineering Toolbox, Emissivity Coefficient Materials, 2003. https://www.engineeringtoolbox.com/emissivity-coefficients-d_447.html (Accessed 12 February 2021).

